# Electroconvulsive therapy (ECT) with ketamine induction for catatonia in an HIV positive patient

**DOI:** 10.4102/sajpsychiatry.v29i0.1944

**Published:** 2023-01-30

**Authors:** Yvette Nel, Craig A. Bracken

**Affiliations:** 1 Department of Psychiatry, Faculty of Health Science, University of the Witwatersrand, Johannesburg, South Africa

**Keywords:** ECT, case report, Ketamine, HIV, catatonia

## Abstract

**Introduction:**

The successful use of ECT as treatment for catatonia, in the context of HIV (human immunodeficiency virus) infection, has been described previously. Ketamine has been used as an anaesthetic induction agent for ECT, although not considered the induction agent of choice. There are also case reports suggesting that ketamine may be an alternative treatment specifically for catatonia.

**Patient presentation:**

This case report describes the management of a female patient who presented with catatonia, evidenced by stupor, waxy flexibility, mutism, negativism, and stereotypy, as well as stage four HIV infection, with poor response to previous psychotherapeutic interventions.

**Management and outcome:**

We describe the course of management of this patient with ECT, following poor initial clinical response to ECT with propofol induction, the subsequent use of ketamine as an anaesthetic induction agent for ECT, with associated improvement in seizure quality, and good overall clinical response to ECT demonstrated thereafter.

**Conclusion and contributions:**

This case report suggests that ketamine may be a viable induction agent for ECT in this clinical setting.

## Introduction

Catatonia is a longstanding recognised indication for electroconvulsive therapy (ECT).^1^ The diagnosis underlying catatonic presentations includes schizophrenia, severe depression and general medical conditions.^1^ The frequency of hospitalisation for catatonia has reportedly decreased since the 1920s, with explanations for this observation including more effective treatment options for medical conditions, as well as for mood and psychotic disorders.^1^ There have been a few case reports of catatonic presentations linked to human immunodeficiency virus (HIV), specifically linked with mania and psychosis, as well as progressive multifocal leukoencephalopathy (PML) and psychosis secondary to antiretroviral medication.^2,3,4,5^ The successful use of ECT in HIV-positive individuals presenting with catatonia has previously been described in case reports.^6,7^

The overall efficacy of ECT has been linked to several factors, including seizure quality as indicated by, firstly, seizure duration over 25 s; secondly, heart rate response indicating a seizure-related sympathetic response; and thirdly, overall seizure quality as observed from the electroencephalogram (EEG) pattern of progression from low amplitude polyspike to higher amplitude spike and wave before postictal suppression.^8^ The choice of anaesthetic induction agent may directly impact the overall seizure quality and outcome of ECT; propofol, for example, has anticonvulsant properties compared to etomidate, and may therefore worsen overall seizure quality; sevoflurane has also been shown to decrease seizure duration.^9^ Ketamine is a dissociative anaesthetic agent which is widely used in a variety of settings for analgesia and anaesthesia. The use of ketamine as the first-line choice of induction agent for ECT is theoretically limited due to its side effect profile, which can include dissociative properties, with the occurrence of dose-dependent perceptual disturbances and delirium-like states on emergence from anaesthesia, as well as concerns around increasing the risk of arrythmias.^10^ However, ketamine has been used successfully in anaesthesia for ECT, with noted improved seizure duration, and has been well tolerated.^11,12^ There are also a few case reports suggesting that ketamine may be also an effective alternative treatment for catatonia.^13,14^

This case report describes the successful use of ketamine as an induction agent in ECT for prolonged catatonia in a patient with HIV.

## Ethical considerations

Permission for publication was obtained from the specialised psychiatric hospital and the University of the Witwatersrand Human Research Ethics Committee (reference number M200384). Informed consent for publication of the case report was also obtained from the patient and next of kin.

## Patient presentation

Ms X, a 36-year-old woman, presented to a specialised psychiatric hospital as a referral from a peripheral district psychiatric unit. She was known to the district hospital following an index presentation six years before this current admission, with behavioural disturbance, aggression and mutism. She had been diagnosed with HIV and had been on combined antiretroviral therapy (ART) (fixed oral dose combination of efavirenz, emtricitabine, and tenofovir) for approximately five years. She had also been treated with clozapine 350 mg by mouth at night and citalopram 30 mg by mouth in the morning at the district hospital, although record-keeping was poor, and it was not possible to determine how this combination of psychotropic medication had been reached. The mutism had remained prominent since the first presentation. She was admitted to the district hospital three weeks prior to transfer with deteriorating agitated behaviour, aggression, refusal to eat and ongoing mutism. She had been given a trial of oral lorazepam with no improvement in symptoms. She was known from the referral hospital with a chronic, five year history of nonprogressive raised alkaline phosphatase (ALP) and gamma-glutamyl transferase (GGT), for which preliminary workup had been completed prior to transfer, including a normal ultrasound scan of the abdomen.

On arrival at the hospital, she was noted to be mute, uncooperative and combatively refusing to be examined by avoiding gaze and interaction and resisting movement. Physically, she had a body mass index (BMI) of 30 kg/m^2^ and a scar from previous herpes zoster over her right forehead. She was refusing food in the ward, did not interact with other patients or staff and spent most of her time in bed. It was not possible to assess thought content or mood, nor to complete any neurocognitive screening. Catatonia was diagnosed clinically based on the presence of the following DSM-5 criteria: (1) stupor, (2) waxy flexibility, (3) mutism, (4) negativism and (5) stereotypy.

Blood workup demonstrated advanced HIV infection (Stage 3 HIV infection, HIV viral load 106 copies/ml, CD4 count was 193 cells/mm^3^) with no other significant abnormalities noted, other than ongoing, nonprogressive raised ALP and GGT. Lumbar puncture results were within normal limits. An electroencephalogram performed two weeks after admission showed generalised slowing (theta rhythm). Recent computerised tomography of the brain (CTB) from the referral hospital showed patchy leukomalacia, suggesting chronic lacunar infarcts, consistent with previous CTB five years earlier.

## Management and outcome

There was no clinical or laboratory cause found for the EEG and CTB scan findings, and the magnetic resonance imaging (MRI) booking date was several months away. Work-up for possible ECT, including repeat urea and electrolytes and ECG, as well as repeat liver function tests with no worsening of the ALP and GGT, showed no contraindications to performing ECT, and therefore it was decided to commence with a trial of ECT.

Details of 10 ECT treatments are provided in Table 1. Bitemporal ECT was administered twice a week. The decision to use ketamine as an induction agent from session seven was based on a lack of availability of etomidate, which was the preferred agent, and the documented poor seizure quality with propofol induction. Following the decision to use ketamine as the induction agent, combined with a decision to double the dosage of ECT at session seven, the improvement in seizure quality on several parameters (duration of seizure on clinical, EEG and EMG recordings as well as heart rate response) was noted. There was no record of the emergence of delirium or perceptual disturbances. There was a significant improvement noted immediately post session eight, with the resumption of spontaneous speech on interview 2 h post ECT, a reactive affect and improved interaction. The improvement continued and was sustained for two further sessions of ECT, with subsequent termination of ECT. There was a sustained improvement for 6 weeks post ECT. The patient was discharged to follow-up at the district hospital on quetiapine 300 mg at night, citalopram 40 mg daily and a fixed dose combination of antiretrovirals (tenofovir, emtricitabine and efavirenz). Neurocognitive testing post ECT was limited due to language and educational constraints. However, functional improvement in several symptom domains was noted by the clinical team, as well as positive feedback regarding a significant recovery from the patient’s relatives.

**TABLE 1:** Summary of electroconvulsive therapy.

ECT	% energy delivered	Pulse width (ms)	Impedance (Ohm)	Anaesthetic agents used	Duration of seizure (seconds)			Clinical parameters of seizure		Clinical response post ECT
					Clin	EEG	EMG	max HR	PSI %	
1	15%	0.5	750	Etomidate 16 mg	24	24	26	NR	54.8	nil
2	15%	0.5	800	Etomidate 20 mg	13	NR	NR	NR	NR	nil
	30%	0.5	800		21	NR	NR	NR	NR	
3	30%	0.5	1330	Etomidate 16 mg	26	26	26	124	NR	nil
4	45%	0.5	1020	Propofol 160 mg	6	8	8	NR	NR	nil
	90%	-	-		8	9	9	104	75.2	
5	100%	0.5	1360	Propofol 80 mg	9	26	11	108	50.8	nil
6	100%	0.5	1150	Propofol 190 mg	7	13	5	91	89	Improved eye contact
	100%	0.5	1010		7	8	5	92	67.6	
7	200%	1.0	1180	Ketamine 50 mg	NR	28	26	153	NR	Mute, eating well, cooperative
8	200%	1.0	1070	Ketamine 100 mg	24	39	23	154	83.3	Normal spontaneous speech, fatuous affect
9	200%	1.0	810	Ketamine 100 mg	25	27	21	120	94.3	Euthymic, normal speech, good contact, apsychotic
10	200%	1.0	1340	Ketamine 100 mg	21	16	13	133	NR	Maintaining response, interacting well, normal speech

NR, not recorded; PSI, postictal suppression index; HR, heart rate; ECT, electroconvulsive therapy; EEG, electroencephalogram; EMG, electromyogram; Clin, clinically observed seizure duration.

## Discussion

Ketamine is an N-methyl-D-aspartate (NMDA) receptor antagonist, and theories regarding its efficacy in depression relate to its role in glutamate signalling and synaptic plasticity.^15^ The pathophysiology of catatonia remains unclear. There are suggested abnormalities in dopamine, glutamate and gamma-aminobutyric acid (GABA) activity in patients with catatonia.^16^ Some evidence suggests that catatonia may be a manifestation of extreme anxiety, even though this is not universally reported.^17^ Theoretical explanations of the direct benefit of ketamine in catatonia have not been well described.

There are case reports of successful treatment of catatonia in HIV-positive individuals using ECT.^6,7^ However, the long duration of mutism as well as the underlying advanced HIV and CT scan findings suggesting chronic irreversible cerebral damage in this individual made the prospect of successful treatment seem unlikely. The hesitancy to commence ECT by the treating team was rooted in the belief that ECT would probably be of no benefit, as well as anxiety in searching for another reversible or treatable cause for delirium, other than advanced HIV, although the positive response to ECT for protracted delirium has previously been described.^18^ The initial poor response to the first six sessions of ECT was noted and seemed to confirm the unlikely prospect of successful treatment. The subsequent significant clinical improvement in clinical symptoms after session eight and resolution of catatonia, however, still came as a surprise to the treating team.

There are three possible explanations for the response after session eight. Firstly, it is possible that the use of ketamine lowered the seizure threshold and, in addition to the increased treatment dose to deliver the ECT, resulted in adequate seizure quality which led to a subsequent clinical response; this hypothesis is supported by the resolution of catatonia after two sessions where adequate seizure (duration and heart rate response) was observed (session seven and eight). Secondly, it is possible that the use of ketamine, regardless of the adequate seizure, at a dose of > 0.5 mg/kg had a direct causal effect on the improvement of catatonia, although the improvement was only noted immediately after the second dose of ketamine was given. Thirdly, it is possible that both the improvement in seizure quality (seizure duration and heart rate response) and direct effect of ketamine synergistically resulted in an improvement in catatonia.

Evidence supporting the use of ketamine in psychiatry is inconsistent. While there is evidence to suggest rapid improvement in depressive symptoms following ketamine infusions, the risk of dependence and abuse, as well as the initial euphoria associated with ketamine, remain concerns, as does the inability to fully ‘mask’ the immediate effects of ketamine in controlled studies.^19^ There are also a few case reports of the successful use of ketamine infusions in the treatment of catatonia but no larger comparative studies.^13,14^ Cobb and Nanda note a lack of high-quality evidence to suggest that ECT with ketamine is superior to ECT with other anaesthetic agents, and they also report limited evidence regarding the superiority of the direct effects of ketamine on depression when compared to ECT.^10^

There are a few case reports of liver dysfunction associated with regular recreational ketamine use in HIV-positive men on ART, specifically ritonavir-containing regimes.^20^ Ketamine is metabolised primarily by CYP2B6, as well as CYP3A4 and CYP2C9. Ritonavir is a potent CYP2B6 inhibitor, which may therefore increase the risk of ketamine toxicity.^21^ The use of ART was not considered to be a contraindication to intravenous bolus induction agent anaesthetic use of ketamine in this patient.

## Limitations of this case report

The pathogenesis of catatonia related to advanced HIV, ART treatment or the underlying pathological EEG and CT brain changes in this patient are beyond the scope of this case presentation and has not been further explored. Magnetic resonance imaging was not available. Poor district-level record-keeping regarding the duration of catatonia, symptom progression and associated medical conditions makes a retrospective diagnosis of ‘prolonged catatonia’ uncertain. Raised ALP and GGT were not further investigated. Longer-term follow-up has not been possible to assess for any re-emergence of catatonia and long-term benefit or adverse effects following ECT. Limitations of case reports have previously been described which apply in this case, including that a single case report is not significant evidence for a cause–effect relationship; the case report is retrospective and naturalistic; and the risk of overinterpretation bias exists.^22^

## Conclusion

This case report illustrates the safe and effective use of ketamine as an induction agent for anaesthesia for ECT in an HIV-positive patient presenting with catatonia. Improved seizure quality resulting from the use of ketamine as an induction agent is a possible factor in the positive response to ECT.
